# A rare case of "switch on and off" multi-system Langerhans cell histiocytosis in an adult patient

**DOI:** 10.1186/1752-1947-5-302

**Published:** 2011-07-11

**Authors:** Georgia Karpathiou, Anastasios Koutsopoulos, Marios E Froudarakis

**Affiliations:** 1 Department of Pneumonology, Medical School, Democritus University of Thrace, 68100 Alexandroupolis, Greece

## Abstract

**Introduction:**

We report the case of a 24-year-old Greek woman with histologically proven osseous and pulmonary Langerhans cell histiocytosis whose lesions had progressively regressed with a "switch on and off" mode. This is the first report in the literature of this mode of presentation of Langerhans cell histiocytosis.

**Case presentation:**

The patient had first presented at the age of 20 years with a solitary lesion of the humerus which spontaneously regressed. At that time, no therapy or smoking cessation was indicated. Four years later she presented with bilateral pneumothorax and pulmonary lesions of Langerhans cell histiocytosis. She had pleurodesis for this disease-related complication, and no further systemic treatment was applied, except with regard to smoking cessation. During the follow-up period, her pulmonary lesions regressed without recurrence six years after the initial lung involvement.

**Conclusion:**

This uncommon case of remission of multi-system Langerhans cell histiocytosis indicates the unpredictable evolution of the disease, raising the question of conservative management in such a patient.

## Introduction

Langerhans cell histiocytosis (LCH) is a rare clinicopathologic entity of unknown etiology with variable system involvement, but with the common characteristic of sizable Langerhans cell infiltration [1]. The clinical course, as well as the treatment and prognosis of this disease, are not clearly identified, especially in the adult population [2]. In pulmonary LCH, smoking cessation is mandatory, while glucocorticoid therapy, despite being used, has not been proved to be an effective treatment method [3]. In multi-system LCH, children with aggressive disease are usually treated with multi-agent chemotherapy [4,5]. Remission has been observed in single-system disease after smoking cessation [3,6]. We report a rare case of "switch on and off" remission of multi-system disease with alternative bone and pulmonary manifestations in an adult patient after only smoking cessation.

## Case presentation

**Figure 1:**
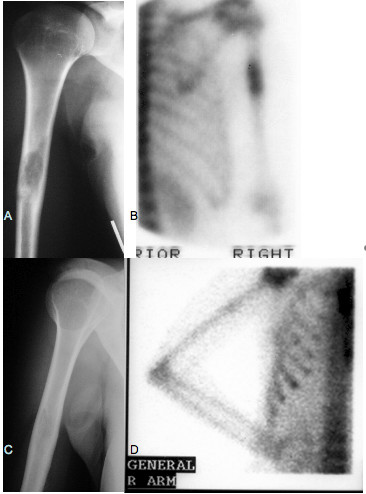
**(A) Radiography and (B) bone scan of the right humerus at initial diagnosis showing Langerhans cell histiocytosis localization on the one-third medium which resolved (C and D) at the time of pulmonary manifestation**.

A 24-year-old Greek woman was referred to our hospital with bilateral pneumothorax. The patient's symptoms had started four years previously, when she consulted her personal physician for persistent pain of the right humerus. The diagnostic approach at the time revealed a lesion detected by radiography of the right humerus and confirmed by a bone scan (Figures 1A and 1B), for which the patient underwent only a biopsy, and no surgical resection or osseous curettage was performed. Microscopic examination revealed osseous LCH, and neither further treatment nor follow-up was recommended. At the time, she was an occasional smoker with normal chest radiography and high-resolution chest tomography (HRCT) results, and no smoking cessation was advised.

**Figure 2:**
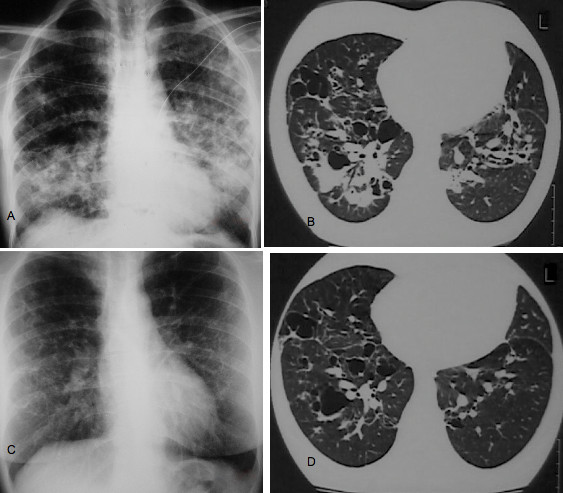
**(A) Radiography of the chest at the time of bilateral pneumothorax revealing pulmonary Langerhans cell histiocytosis**. **(B) **High-resolution computed tomography (HRCT) of the chest showing multiple cysts associated with nodules and extensive consolidation. **(C) **Radiography of the chest and **(D) **HRCT one year later showing significant improvement of the initial findings and regression of consolidation.

**Figure 3:**
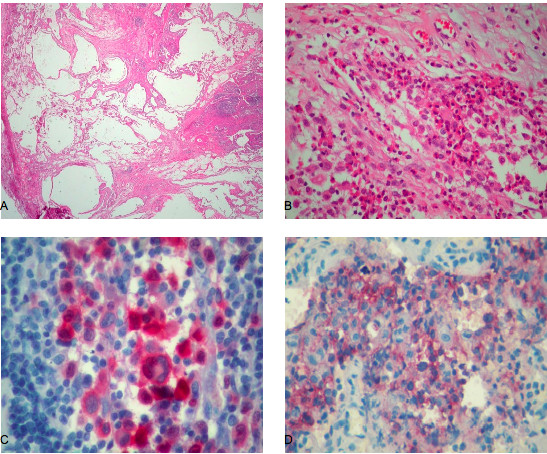
**(A) Tissue slide revealing an obvious distortion of lung architecture (hematoxylin and eosin stain; original magnification, ×20)**. **(B) **Higher magnification (original magnification, ×200) of the slide in Figure 3A showing the characteristic ovoid Langerhans cells and eosinophils (hematoxylin and eosin stain). **(C) **The Langerhans cells demonstrate strong immunoreactivity, both nuclear and cytoplasmic, for S-100 protein (original magnification, ×400). **(D) **CD1a stain is also positive in Langerhans cells (original magnification, ×400).

The patient was symptom-free for the following four years, at which point she was admitted to our hospital for the treatment of bilateral, spontaneous pneumothorax (Figure 2A) and diffused interstitial lung disease. During the preceding four-year period, she had increased her cigarette smoking to one pack per day. She complained of no symptoms, such as cough, dyspnea or fever. HRCT showed multiple cysts and nodules as well as extensive consolidation (Figure 2B). A mild inflammatory syndrome was observed on the basis of biological examination. Biopsies taken during a thoracoscopy performed for left pleurodesis showed distortion of the lung architecture and infiltration by Langerhans histiocytes (Figures 3A and 3B). She tested positive for S-100 protein and CD1a (Figures 3C and 3D). Her lung function tests, performed one month after the resolution of her pneumothoraces, showed a restrictive syndrome with decreased static volumes and normal diffusion capacity factor. The results of her lung function tests were as follows: forced vital capacity (FVC) = 2.16 L (63.3%), forced expiratory volume in 1 second (FEV_1_) = 1.76 L (58.9%), FEV_1_/FVC = 81.3% (95.9%), transfer coefficient for carbon monoxide (TLCO)/alveolar volume (VA) = 2 mmol/min/kPa/L (93.3%), residual volume (RV) = 1.03 L (86.8%), total lung capacity (TLC) = 3.23 L (71.7%) and functional residual capacity (FRC) = 1.65 L (65.7%). A new radiographic examination and bone scan showed the extinction of the lesion on the right humerus (Figures 1C and 1D). We decided to stop treatment, except for advising the patient to cease smoking immediately, which she did.

One year later, during her follow-up examination, her chest X-ray and HRCT (Figures 2C and 2D) were impressively improved. In addition, improvement was noted in her static lung volumes: FVC = 2.3L (67.1%), FEV_1 _= 1.8L (60.3%), FEV_1_/FVC = 78.4% (92.5%), TLCO/VA = 2.05 mmol/min/kPa/L (95.6%), RV = 1.20L (100%), TLC = 4.08L (90.6%) and FRC = 2.05L (81.5%). To date, after six years of follow-up, the patient has had no signs or symptoms of disease activity, and her chest X-rays and lung function tests remain stable.

## Discussion

Our patient had multi-system disease involving alternatively bone and lung. The rarity of this case is that this patient showed remission of her first LCH bone lesion while she did not interrupt smoking. She developed secondary pulmonary disease, which also regressed, after smoking cessation. This case report describes for the first time in the literature a "switch on and off" mode of evolution of LCH.

The clinical course of the LCH varies, while its treatment, outcome and prognosis cannot be precisely defined, since, owing to its relative rarity, and large, randomized, prospective studies of this disease are lacking, especially those concerning adult patients [2,3,7]. Randomized studies performed to date have included children, whereas the available data concerning the origin of LCH in adult patients have been reported mainly in retrospective studies [2]. However, the results of trials performed with children cannot be completely applied to adults, as there are differences in the course of the disease. In a large cohort of patients, children and adults studied retrospectively [8], 30.6% had LCH involving more than one body system, and all of these patients were treated with surgery, chemotherapy or combination therapy. The majority of these treated patients relapsed and underwent second-line treatment [8].

In data collected from the International Histiocyte Society registry, comprising adult patients, single-system disease was found in 31% of the patients and 69% had multi-system disease [7]. The data from that report supported the great uncertainty and lack of a therapeutic standard at least for front-line treatment [2]. In general, multi-system disease is being treated with prednisone, single-agent chemotherapy or multi-agent chemotherapy, according to studies performed in children [2,4,5], whereas in pulmonary LCH, smoking cessation is mandatory, with glucocorticoid therapy used on the basis of no evidence-based data [3]. In another retrospective study of 102 adult patients with pulmonary LCH [9], treatment included no interventions, except for smoking cessation in patients with minimal or no symptoms, prednisone alone or in combination with other immunosuppressive agents was prescribed for 54 patients, while two patients underwent surgical pleurodesis shortly after the diagnosis and one patient underwent lung transplantation at the time of follow-up. In that study in adults with pulmonary LCH, no specific interventions showed any benefit with regard to patient survival [9].

Patients with pulmonary LCH may recover spontaneously or remain in stable condition without treatment [3]. Although pulmonary LCH is usually a single-system disease in adults, the presence of a bone lesion as an extrapulmonary manifestation is not so rare [3]. Spontaneous regression of bone lesions has also been observed in adults [6] as well as in infants [10]. Also, in other diffuse interstitial lung diseases of unknown origin, such as sarcoidosis, the occurrence of pneumothorax does not necessarily lead to steroid therapy [11]. In our patient with LCH, no systemic treatment was applied for the disease. Pleurodesis was performed for pneumothorax, a disease complication, as indicated for patients with spontaneous secondary pneumothorax [12-14]. At the same time, she was advised to cease smoking, which she did. Her pulmonary lesions had secondarily regressed, and the patient is free of relapse to date.

In conclusion, in our patient, LCH osseous and pulmonary lesions regressed with a rare "switch on and off" mode. This uncommon case of remission of multi-system LCH indicates the unpredictable evolution of the disease, raising the question of conservative management in such a patient.

## Consent

Written informed consent was obtained from our patient for the publication of this case report and any accompanying images. A copy of the written consent is available for review by the Editor-in-Chief of this journal.

## Competing interests

The authors declare that they have no competing interests.

## Authors' contributions

Our patient was admitted under the care of MF during this episode and was followed up in an outpatient setting. All authors contributed equally in writing the manuscript. GK wrote the manuscript, AK provided the histological images and MF corrected and refined the manuscript. All authors read and approved the final manuscript.
